# Two-stage variable-fidelity modeling of antennas with domain confinement

**DOI:** 10.1038/s41598-022-20495-y

**Published:** 2022-10-14

**Authors:** Anna Pietrenko-Dabrowska, Slawomir Koziel, Lukasz Golunski

**Affiliations:** 1 Faculty of Electronics, Telecommunications and Informatics, Gdansk University of Technology, 80-233 Gdansk, Poland; 2 Present Address: Engineering Optimization & Modeling Center, Reykjavik University, 102 Reykjavik, Iceland

**Keywords:** Electrical and electronic engineering, Computational science

## Abstract

Surrogate modeling has become the method of choice in solving an increasing number of antenna design tasks, especially those involving expensive full-wave electromagnetic (EM) simulations. Notwithstanding, the curse of dimensionality considerably affects conventional metamodeling methods, and their capability to efficiently handle nonlinear antenna characteristics over broad ranges of the system parameters is limited. Performance-driven (or constrained) modeling frameworks may be employed to mitigate these issues by considering a construction of surrogates from the standpoint of the antenna performance figures rather than directly geometry parameters. This permits a significant reduction of the model setup cost without restricting its design utility. This paper proposes a novel modeling framework, which capitalizes on the domain confinement concepts and also incorporates variable-fidelity EM simulations, both at the surrogate domain definition stage, and when rendering the final surrogate. The latter employs co-kriging as a method of blending simulation data of different fidelities. The presented approach has been validated using three microstrip antennas, and demonstrated to yield reliable models at remarkably low CPU costs, as compared to both conventional and performance-driven modeling procedures.

## Introduction

Design of contemporary antenna structures is to a large degree based on full-wave electromagnetic (EM) simulation models^1–4^, which are used for the development of antenna topology^5^, parametric studies^6^, as well as geometry adjustment^7,8^. The need for EM analysis arises from the fact that alternative representations (e.g. parameterized equivalent networks) are either non-reliable or non-existent. Moreover, EM simulations reliably account for mutual coupling, the effects of housing, connectors, etc. Furthermore, antenna structures become increasingly sophisticated to realize the assumed functionalities (multi-band and MIMO operation, circular polarization, etc.)^9–13^. To enable these, a variety of topological alterations are incorporated (stubs, defected grounds structures and so on)^14–17^, all of which have to be properly dimensioned. EM analysis is CPU intensive, which impedes execution of EM-driven procedures that entail repetitive simulations, such as optimization^18^, statistical analysis^19,20^, design centering^21,22^, let alone global^23^ or multi-objective search^24^, especially for complex devices^25–27^.

Accelerating EM-based procedures is a matter of practical necessity. Numerous techniques have been developed for that purpose. In the realm of local optimization, we have adjoint sensitivities^28^, mesh deformation^29^, selective gradient updates^30,31^, or the employment of customized EM solvers^32^. In a broader context, surrogate-assisted approaches has been growing in popularity, both concerning physics-based models (space^33^ or manifold mapping^34^, shape-preserving response prediction^35^, or adaptive response scaling^36^), and data-driven ones, e.g. kriging^37^, artificial neural networks (ANN)^38^, radial-basis functions (RBF)^39^, support vector regression (SVR)^40^, Gaussian process regression (GPR)^41^, or polynomial chaos expansion^42^. The latter are frequently used in global and multi-criterial optimization^43–45^. Among other noteworthy methods feature-based optimization (FBO)^46–48^ and cognition-based design^49^, in which the design task is re-mapped into the space of adequately pinpointed attributes (points) of the system outputs, the latter being in weakly nonlinear relationship with the geometry parameters.

Needless to say, replacing EM analysis by fast surrogates is invaluable as an acceleration tool. Approximation models (kriging^50^, RBF^51^, PCE^52^, SVR^53^, ANNs and numerable variants thereof, e.g. convolutional neural networks (CNN)^54^ or deep neural networks (DNN)^55^) are particularly popular due to their accessibility^56,57^. However, data-driven modeling methodologies are largely influenced by the curse of dimensionality and at the same time, have limited capability to represent highly nonlinear antenna characteristics. Available mitigation tools, e.g. least-angle regression (LAR)^58^, high-dimensional model representation (HDMR)^59^, are not suitable for general-purpose antenna modeling. Meanwhile, variable-resolution methods have been demonstrated to be beneficial in this context (co-kriging^60^, two-level GPR^61^), also in combination with sequential sampling^62–65^.

Recent performance-driven (or constrained) modeling^66^ proposes a different manner of alleviating the problems of standard techniques by limiting the metamodel domain to a small district that contains increased-quality designs (w.r.t. the assumed figures of interest, e.g. operating frequencies). Domain confinement remarkably lowers computational expenditures of acquiring the training data without restricting the design utility of the model^67^. Constrained modeling comes in several variations that incorporate, among others, dimensionality reduction and variable-fidelity EM models^68–71^. The fundamental problem of the aforementioned techniques is an inflated initial cost associated with identification of the database designs, otherwise necessary to set up the domain of the surrogate^68^. To some extent, this issue can be mitigated by involving sensitivity information^72^. In^73^, an alternate way to performance-driven modeling has been introduced, which abandons the use of reference designs in favor of random trial points. Information extracted therefrom is used to define the domain with the use of an auxiliary inverse regression model. The method of^73^ has been shown to retain the benefits of constrained modeling which reduces the initial cost by almost seventy percent in some cases.

In this paper, we propose a novel antenna modeling approach, which employs the performance-driven paradigm, and advances over the reference-design-free approach presented in^73^ by exploiting variable-resolution EM simulations. More specifically, generation of the trial points, necessary to identify the inverse regression model, is executed at the level of a coarse-discretization EM simulations. Furthermore, the majority of the training data points acquired to build the final metamodel are obtained at the same level, and supplemented by a small amount of high-fidelity samples. The data of both resolution levels is then blended using co-kriging^74^. Numerical validation performed using three antenna structures demonstrates that our framework allows to achieve additional computational savings of up to 64 percent over the technique of^73^, and as high as 82 percent over the nested kriging framework^68^ with regard to the model setup costs. This speedup is obtained without degrading the predictive power of the surrogate. Moreover, it ensures a remarkable accuracy improvement over conventional modeling methods.

### Two-level variable-fidelity modeling within restricted domain

This section formulates the modeling methodology proposed in the paper. First, the concept of performance-driven modeling is recalled, along with an outline of the trial point acquisition (section “Performance-driven modeling basics”). In sections two-stage modeling: trial points and inverse regression model and surrogate domain definition, inverse regression model and surrogate domain definition are delineated, respectively. Section final surrogate. variable-resolution models and co-kriging discusses a construction of the final model using co-kriging, whereas section modeling procedure summary summarizes the complete procedure.

### Performance-driven modeling basics

Here, we recall the basics of constrained (or performance-driven) modeling. The main concept is to construct the surrogate in a small portion of the parameter space, which encloses the designs being nearly optimum with regard to the target objective vectors. The computational benefits are due to a small volume of such a subset and a smaller sizes of training data sets that are needed to construct model of a satisfactory predictive power^75^.

Table 1 outlines the notation used by constrained modeling frameworks^68^. The figures of interest may include antenna operating frequencies (but also the bandwidths or permittivity of a dielectric substrate). The central idea here pertains to the objective space *F* that determines the validity zone of the metamodel. Another important entity is the model domain. Its establishment involves the concept of design optimality, that is measured using a merit function *U*(***x***, ***f***)^69^. The solution x^*^, which is optimal with respect to a performance vector ***f*** ∈ *F*, is given as

1 $${\mathbf{x}}^{*} = U_{F} ({\mathbf{f}}) = \arg \mathop {\min }\limits_{{\mathbf{x}}} U({\mathbf{x}},{\mathbf{f}}).$$

**Table 1:** Notation used in performance-driven modeling.

Description	Notation
Antenna parameter vector	***x*** = [*x*_1_ … *x*_*n*_]^*T*^
Conventional parameter space	*X* = [***l***, ***u***]
Lower bounds on the parameters	***l*** = [*l*_1_ …, *l*_*n*_]^*T*^
Upper bounds on the parameters	***u*** = [*u*_1_ …, *u*_*n*_]^*T*^
Figures of interest	*f*_*k*_, *k* = 1, …, *N*
Objective space	*F*: *f*_*k.*min_ ≤ *f*_*k*_^(*j*)^ ≤ *f*_*k.*max_, *k* = 1, …, *N*
Objective vector	***f*** = [*f*_1_ … *f*_*N*_]^*T*^

The optimum design manifold, consisting of designs (1) obtained for all ***f*** ∈ *F*, constitutes an *N*-dimensional object in *X*, given as

2 $$U_{F} (F) = \left\{ {U_{F} ({\mathbf{f}}):{\mathbf{f}} \in F} \right\}.$$

**Figure 1:**
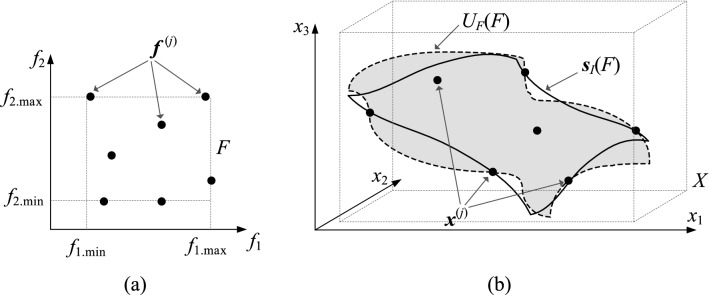
Basics of confined modeling^68^: (**a**) the objectives’ space *F*, (**b**) design space *X* (black circles mark the trial points, grey surface indicates the manifold of optimum designs *U*_*F*_(*F*)). The image of the first-layer metamodel ***s***_*I*_(*F*) yields a rough assessment of the manifold, so it needs to be outstretched to encompass entire *U*_*F*_(*F*).

The domain of the surrogate model is determined as a neighborhood of *U*_*F*_(*F*)^69^. In a majority of performance-driven frameworks, its definition exploits database designs ***x***^(*j*)^ = [*x*_1_^(*j*)^ … *x*_*n*_^(*j*)^]^*T*^, *j* = 1, …, *p*, optimal with respect to (1) for objective vectors ***f***^(*j*)^ = [*f*_1_^(*j*)^ … *f*_*N*_^(*j*)^], distributed in *F*. More specifically, the couples {***f***^(*j*)^,***x***^(*j*)^}, *j* = 1, …, *p*, serve as a training points to build the first-layer model ***s***_*I*_(***f***) : *F* → *X* ^68^ that approximates *U*_*F*_(*F*), see Fig. 1. The metamodel domain itself constitutes an orthogonal extension of the first-level surrogate^69^.

Acquisition of the database designs is a bottleneck of performance-driven modeling procedures because it requires a large number (a few hundreds to over a thousand) of antenna simulations^73^. These extra expenses contribute to the overall model setup cost. As mentioned earlier, the number of trial points can be reduced by exploiting the sensitivity information^72^, whereas their identification costs may be diminished by warm-start optimization algorithms (e.g.^76^).

The recent constrained modeling framework^73^ introduced an alternative method for defining the model domain, which utilizes random trial points in place of the reference designs. The details of this method will be recalled in section two-stage modeling: trial points and inverse regression model, as it is one of the constituent parts of the introduced modeling procedure proposed.

### Two-stage modeling: trial points and inverse regression model

First, we will define the surrogate model domain. Toward this end, we need to roughly assess the optimum design manifold *U*_*F*_(*F*) of Eq. (2). We follow the methodology of^73^, where the required information is extracted from a set of random trial points, allocated in the design space *X*.

**Figure 2:**
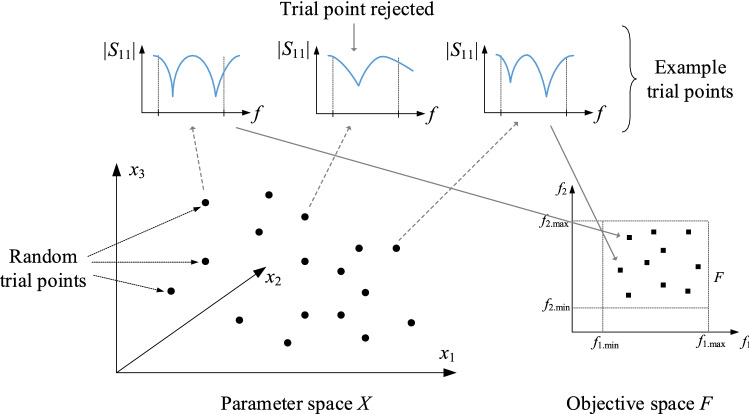
Procedure of the random trial point rendition for an exemplary dual-band antenna (the objective and parameter space are two- and three-dimensional, respectively). The random designs whose resonant frequencies reside within the required confines of the objective space are retained, the remaining ones are discarded. The inverse regression model *s*_*r*_(⋅) is built using the ultimate trial point set {***x***_*r*_^(*j*)^}_*j* = 1,…,*Nr*_.

We denote by ***x***_*r*_^(*j*)^ = [*x*_*r.*1_^(*k*)^ … *x*_*r.n*_^(*k*)^]^*T*^, *j* = 1, 2, … a series of random trial points yielded according to a uniform probability distribution and by ***f***_*r*_^(*j*)^ = [*f*_*r.*1_^(*j*)^ … *f*_*r.N*_^(*j*)^]^*T*^ the performance figure vectors derived from the antenna response rendered by the full-wave model at ***x***_*r*_^(*j*)^. The performance figures are the same as the elements of the objective space *F*, e.g. the antenna resonant frequencies. The trial point is accepted if the extracted ***f***_*r*_^(*j*)^ resides in *F* and rejected otherwise (i.e. if the entries of ***f***_*r*_^(*j*)^ are located outside the lower and upper limits *f*_*k.*min_ and *f*_*k.*max_, cf. Table 1, or if ***f***_*r*_^(*j*)^ cannot be identified due to distorted antenna responses). Figure 2 gives a graphical illustration of the trial point selection for an exemplary dual-band antenna. The process of generating the trial points continues until the assumed number *N*_*r*_ (e.g. 50) has been identified. For each accepted point ***x***_*r*_^(*j*)^, an additional vector ***p***_*r*_^(*j*)^ = [*p*_*r.*1_^(*j*)^ … *p*_*r.M*_^(*j*)^]^*T*^ is extracted from antenna responses. The said vector ***p***_*r*_^(*j*)^ contains the information pertaining to antenna performance. In our example, these may refer to the reflection coefficient levels at the resonant frequencies, in which case *M* = *N*.

Using the set {***x***_*r*_^(*j*)^,***f***_*r*_^(*j*)^,***p***_*r*_^(*j*)^}_*j* = 1,…,*Nr*_, an inverse regression metamodel *s*_*r*_: *F* → *X* is constructed, serving as a rough assessment of the manifold *U*_*F*_(*F*). The analytical form of the model is taken as (cf.^73^)

3 $$s_{r} ({\mathbf{f}}) = {\mathbf{s}}_{r} \left( {[f_{1} \;...\;f_{N} ]^{{\text{T}}} } \right) = \left[ \begin{gathered} s_{r.1} ({\mathbf{f}}) \\ \cdots \\ s_{r.n} ({\mathbf{f}}) \\ \end{gathered} \right] = \left[ \begin{gathered} a_{1.0} + a_{1.1} \exp \left( {\sum\nolimits_{k = 1}^{N} {a_{1.k + 1} f_{k} } } \right) \\ \cdots \\ a_{n.0} + a_{n.1} \exp \left( {\sum\nolimits_{k = 1}^{N} {a_{n.k + 1} f_{k} } } \right) \\ \end{gathered} \right].$$

The rationale for adopting the specific analytical form of the inverse surrogate as in Eq. (3) is that it is sufficient to adequately reflect typically weakly-nonlinear relationships between the antenna geometry and operating parameters. Moreover, exponential terms feature only few parameters, and are flexible in the sense that they allow for modeling various curvatures, e.g. inverse proportionality occurring for certain parameters.

The coefficients of the inverse surrogate are found as the solutions of the following tasks.

4 $$\left[ {a_{j.0} \;a_{j.1} \;...\;a_{j.N + 1} } \right] = \arg \mathop {\min }\limits_{{[b_{0} \;b_{1} \;...\;b_{K + 1} ]}} \sum\limits_{k = 1}^{{N_{r} }} {w_{k} \left[ {s_{r.j} \left( {{\mathbf{f}}_{r}^{(k)} } \right) - x_{r.j}^{(k)} } \right]^{2} } ,\,{\text{j}}\,{ = }\,{1,} \ldots {\text{,n}}{.}$$

**Figure 3:**
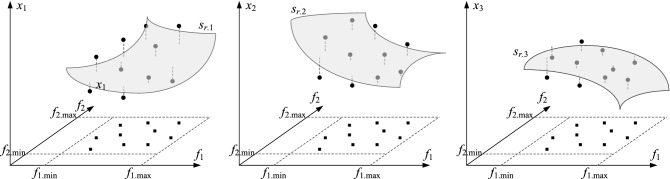
Inverse regression model *s*_*r*_ constructed based on the pre-selected trial points ***x***_*r*_^(*j*)^ along with their respective objectives ***f***_*r*_^(*j*)^. The model elements *s*_*r.j*_ are are shown as grey surfaces for the parameters *x*_1_ (left), *x*_2_ (middle), and *x*_3_ (right), respectively.

The weigh factors *w*_*k*_ are computed as

5 $$w_{k} = \left[ {W - \max \{ p_{1} ({\mathbf{x}}^{(j)} ),...,p_{N} ({\mathbf{x}}^{(j)} )\} } \right]^{2} ,\,{\text{k}}\,{ = }\,{1,} \ldots {\text{,N}}_{r} ,$$

where *W* = max{*k* = 1,…,*N*_*r*_, *j *= 1,…,*N*: *p*_*j*_^(*k*)^}. Here, we assume that *p*_*j*_^(*k*)^ > 0, and better designs are associated with lower values of *p*_*j*_^(*k*)^. The factors *w*_*k*_ are introduced to promote high-quality trial points so as to increase their contribution to the inverse model, which is because these vectors are located in a closer proximity of *U*_*F*_(*F*). Figure 3 shows graphically the concepts of the model *s*_*r*_ in accordance with the example of Fig. 2. Observe that the design space needs to be selected with a proper consideration with respect to the anticipated design quality therein, i.e. the ranges of the antenna geometry parameters have to be established reasonably. Otherwise, the number of trial points necessary to gather the assumed number of the observables of decent quality may be too excessive and thus, the computational benefits of the introduced technique may be compromised. In this work, we assume that the design space has been selected using at least a rudimentary problem-specific knowledge.

### Surrogate domain definition

As discussed earlier, the set *s*_*r*_(*F*) approximates the manifold *U*_*F*_(*F*). The metamodel domain should contain a possibly large part of *U*_*F*_(*F*) so as to account for the designs being optimum (or close to optimum) with respect to all ***f*** ∈ *F*. In^73^, this is achieved by orthogonally extending *s*_*r*_(*F*) in all directions orthogonal to this set. Let us define a orthonormal basis of vectors normal to *s*_*r*_(*F*) at ***f*** as {***v***_*n*_^(*k*)^(***f***)},* k* = 1,…,*n–N*. Let also ***T*** = [*T*_1_ … *T*_*n*_]^*T*^ denote a vector of non-negative extension factors. Further, let us compute the coefficients of the extension

6 $${{\varvec{\upalpha}}}({\mathbf{f}}) = [\alpha_{1} ({\mathbf{f}})\;...\;\alpha_{n - N} ({\mathbf{f}})]^{{\text{T}}} = \left[ {|{\mathbf{Tv}}_{n}^{(1)} ({\mathbf{f}})|\;\;...\;\;|{\mathbf{Tv}}_{n}^{(n - N)} ({\mathbf{f}})|} \right]^{{\text{T}}} .$$

Using (6), the metamodel domain *X*_*S*_ is given as

7 $$X_{S} = \left\{ \begin{gathered} {\mathbf{x}} = s_{r} ({\mathbf{f}}) + \sum\limits_{k = 1}^{n - N} {\lambda_{k} \alpha_{k} ({\mathbf{f}}){\mathbf{v}}_{n}^{(k)} ({\mathbf{f}})} :{\mathbf{f}} \in F,\; \hfill \\ \;\;\;\;\; - 1 \le \lambda_{k} \le 1,\;k = 1,...,n - N \hfill \\ \end{gathered} \right\}.$$

The meaning of this definition is that *X*_*S*_ comprises of all designs given by (7), generated for every possible combination of the objective vectors from the space *F* and *λ*_*k*_ ∈ [−1, 1], *k* = 1,…,*n*–*N*. Observe that the domain lateral bounds are the manifolds $S_{ + } = \left\{ {{\mathbf{x}} \in X:{\mathbf{x}} = s_{r} \left( {\mathbf{f}} \right) + \sum\nolimits_{k = 1}^{n - N} {\alpha_{k} ({\mathbf{f}}){\mathbf{v}}_{n}^{(k)} ({\mathbf{f}})} } \right\}$ and $S_{ - } = \left\{ {{\mathbf{x}} \in X:{\mathbf{x}} = s_{r} \left( {\mathbf{f}} \right) - \sum\nolimits_{k = 1}^{n - N} {\alpha_{k} ({\mathbf{f}}){\mathbf{v}}_{n}^{(k)} ({\mathbf{f}})} } \right\}$.

The extension factors *T*_*j*_ are established individually for all parameters using the trial points {***x***_*r*_^(*j*)^} and the following procedure. We denote the *k*th parameter’s lower and upper bounds as *l*_*k*_ and *u*_*k*_, respectively. Given the pair {***x***_*r*_^(*j*)^, ***f***_*r*_^(*j*)^}, we define *P*_*k*_(***x***_*r*_^(*j*)^) ∈ [*l*_*k*_* u*_*k*_] × *F* as the vector for which the distance between [*x*_*r.k*_^(*j*)^ (***f***_*r*_^(*j*)^)^*T*^]^*T*^ and [*s*_*r.k*_(***f***) ***f***^*T*^]^*T*^, ***f*** ∈ *F*, i.e.

8 $$P_{k} ({\mathbf{x}}_{r}^{(j)} ) = \arg \mathop {\min }\limits_{{{\mathbf{f}} \in F}} ||[x_{r.k}^{(j)} \;({\mathbf{f}}_{r}^{(j)} )^{{\text{T}}} ]^{{\text{T}}} - [s_{r} ({\mathbf{f}})\;{\mathbf{f}}^{{\text{T}}} ]^{{\text{T}}} ||,$$

is minimal; with the orthogonal projection of [*x*_*r.k*_^(*j*)^ (***f***_*r*_^(*j*)^)^*T*^]^*T*^ onto the representation of *s*_*r.k*_ in [*l*_*k*_* u*_*k*_] × *F* denoted as *P*_*k*_(***x***_*r*_^(*j*)^). Consequently

9 $$d_{r.k} ({\mathbf{x}}_{r}^{(j)} ) = ||[x_{r.k}^{(j)} \;({\mathbf{f}}_{r}^{(j)} )^{{\text{T}}} ]^{{\text{T}}} - [s_{r} (P({\mathbf{x}}_{r}^{(j)} ))\;P({\mathbf{x}}_{r}^{(j)} )^{{\text{T}}} ]^{{\text{T}}} ||,$$

refers to the minimal distance between the said image and [*x*_*r.k*_^(*j*)^ (***f***_*r*_^(*j*)^)^*T*^]^*T*^ (note that *d*_*r.k*_ may be viewed as the distance between the entries of the trial vector and the gray surfaces of Fig. 3). Based on these considerations, we define the extension factors *T*_*k*_ as

10 $$T_{k} = \frac{1}{{2N_{r} }}\sum\limits_{j = 1}^{{N_{r} }} {d_{r.k} ({\mathbf{x}}_{r}^{(j)} )} .$$

Thus, *T*_*k*_ constitutes a half of the averaged distance from the trial point entry to the corresponding image of *s*_*r.k*_. The value of the factor 0.5 is chosen because many trial points are of poor quality and using (full) average distance would lead to excessively large domain containing too many designs that are far from the optimum ones^73^.

### Final surrogate. variable-resolution models and co-kriging

One of the important mechanisms employed in this work to lower the cost of surrogate model construction are variable-resolution EM models. In most cases, the modeling process is executed at a single level of fidelity, except situations where the lower-resolution model is corrected with the use of a handful of high-fidelity samples (e.g. space mapping^77^, co-kriging^74^). In this work, we employ low-fidelity models to accelerate the process of defining the surrogate model domain, as well as to downsize the training data acquisition cost.

#### Variable-resolution EM models

**Figure 4:**
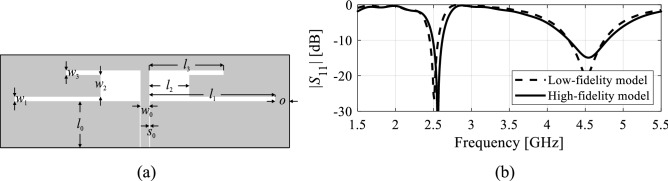
Variable-fidelity models: (**a**) an exemplary dual-band antenna, (**b**) antenna reflection responses evaluated with the low-fidelity EM model (- - -) and the high-fidelity one (—). Here, the high-fidelity model simulation time is about 80 s, whereas the time of the coarse model is only 25 s.

The fundamental benefit of using low-fidelity models, which typically means coarse-discretization EM simulations in the case of antennas^78^, is that the associated simulation time may be considerably shortened, as compared to the high-fidelity version. The latter is normally established to ensure sufficiently accurate rendition of antenna characteristics. The speedup obtained due to low-fidelity analysis is obtained at the expense of the accuracy loss (cf. Fig. 4), which may or may not be problematic, depending on the particular context. For example, in space mapping optimization, the low-fidelity model should to be corrected to make it a reliable prediction tool, at least in the vicinity of the current iteration point^79^. When the low-fidelity model is used to, e.g. parameter space pre-screening, it can usually be used uncorrected^80^.

In this work, the low-fidelity or coarse model, denoted as ***R***_*c*_(***x***), will be used for two purposes: (i) generation of the trial points and metamodel definition as delineated in section two-stage modeling: trial points and inverse regression model, and (ii) accelerating the acquisition of the training data by complementing sparsely-sampled high-fidelity model responses (denoted as ***R***_*f*_(***x***)) with densely-sampled low-fidelity ones. In the case of (ii), co-kriging^74^ is used to combine simulation data of different fidelities. As for (i), because the trial points are only employed to provide an inaccurate approximation of the manifold comprising optimum designs, there is no need to correct a low-fidelity model at this phase of the modeling process, which is advantageous from the standpoint of simplicity of implementation.

#### Final surrogate construction using co-kriging

The final surrogate ***s***_*CO*_(***x***) is generated in the domain *X*_*S*_ using co-kriging^60^. The training data set contains *N*_*Bf*_ high-fidelity pairs {***x***_*Bf*_^(*k*)^,***R***_*f*_(***x***_*Bf*_^(*k*)^)}_*k* = 1, …, *NBf*_, where ***x***_*Bf*_^(*k*)^ ∈ *X*_*S*_, and *N*_*Bc*_ low-fidelity pairs {***x***_*Bc*_^(*k*)^,***R***_*c*_(***x***_*Bc*_^(*k*)^)}_*k* = 1, …, *NBc*_, ***x***_*Bc*_^(*k*)^ ∈ *X*_*S*_. We also use notation *X*_*Bf*_ = {***x***_*Bf*_^(*k*)^}_*k* = 1,…,*NBf*_, and *X*_*Bc*_ = {***x***_*Bc*_^(*k*)^}_*k* = 1,…,*NBc*_.

Let us begin by a recollection of the kriging interpolation, followed by formulation of co-kriging. The kriging surrogate ***s***_*KR*_(***x***) is given as

11 $${\mathbf{s}}_{KR} ({\mathbf{x}}) = {\mathbf{M}}\gamma + r({\mathbf{x}}) \cdot {{\varvec{\Psi}}}^{ - 1} \cdot ({\mathbf{R}}_{f} (X_{Bf} ) - {\mathbf{F}}\gamma ),$$

with ***M*** being a *N*_*Bf*_ × *t* model matrix of *X*_*Bf*_, whereas ***F*** denotes a row vector of the design ***x*** comprising *t* elements (*t* denotes the number of regression function factors^60^); and *γ* refer to coefficients of the regression function

12 $$\gamma = (X_{Bf}^{T} {{\varvec{\Psi}}}^{ - 1} X_{Bf} )^{ - 1} X_{Bf} {{\varvec{\Psi}}}^{ - 1} {\mathbf{R}}_{f} (X_{Bf} ).$$

We also have $r({\mathbf{x}}) = (\psi ({\mathbf{x}},{\mathbf{x}}_{Bf}^{(1)} ),...,\psi ({\mathbf{x}},{\mathbf{x}}_{Bf}^{{(N_{Bf} )}} ))$, constitutes an *N*_*Bf*_—element row vector whose entries are the correlations between *X*_*Bf*_ and ***x***, whereas ***Ψ*** = [Ψ_*i*,*j*_] denotes a correlation matrix, where Ψ_*i*,*j*_ = *ψ*(***x***_*Bf*_^(*i*)^,***x***_*Bf*_^(*j*)^). An exemplary widely used correlation function may be

13 $$\psi ({\mathbf{x}},{\mathbf{x}}^{^{\prime}} ) = \exp \left( {\sum\nolimits_{k = 1}^{n} { - \theta_{k} |x^{k} - x^{^{\prime}k} |^{P} } } \right),$$

where *θ*_*k*_, *k* = 1, …, *n*, are the hyperparameters to be found during model identification, which is achieved through Maximum Likelihood Estimation (MLE)^60^ as

14 $$(\theta_{1} ,...,\theta_{n} ) = \arg \min - (N_{Bf} /2)\ln (\hat{\sigma }^{2} ) - 0.5\ln (|\Psi |),$$

where $\hat{\sigma }^{2} = ({\mathbf{R}}_{f} (X_{Bf} ) - F\alpha )^{T} {{\varvec{\Psi}}}^{ - 1} ({\mathbf{R}}_{f} (X_{Bf} ) - F\alpha )/N_{Bf} ,$ with |***Ψ***| being the determinant of ***Ψ***. In practice, a correlation function of Gaussian type (*P* equal to 2) is often employed, as well as ***M*** = 1 and ***F*** = [1 … 1]^*T*^.

Co-kriging blends together two models: (i) a kriging surrogate ***s***_*KRc*_ set up with the low-fidelity samples (*X*_*Bc*_, ***R***_*c*_(*X*_*Bc*_)) and (ii) the model ***s***_*KRf*_ built using the residuals (*X*_*Bf*_, ***r***), with ***r*** = ***R***_*f*_(*X*_*Bf*_) −* ρ*⋅***R***_*c*_(*X*_*Bf*_). In the latter, *ρ* is a portion of the maximum likelihood estimate of the ***s***_*KRf*_ surrogate. ***R***_*c*_(*X*_*Bf*_) is roughly equal to ***s***_*KRc*_(*X*_*Bf*_). The correlation function of the two models is described by Eq. (13).

The co-kriging model ***s***_*CO*_(***x***) is given by

15 $${\mathbf{s}}_{CO} ({\mathbf{x}}) = {\mathbf{M}}\gamma + r({\mathbf{x}}) \cdot {{\varvec{\Psi}}}^{ - 1} \cdot ({\mathbf{r}} - {\mathbf{F}}\gamma ),$$

where

16 $$r({\mathbf{x}}) = [\rho \cdot \sigma_{c}^{2} \cdot r_{c} ({\mathbf{x}}),\rho^{2} \cdot \sigma_{c}^{2} \cdot r_{c} ({\mathbf{x}},X_{{B_{f} }} ) + \sigma_{d}^{2} \cdot r_{d} ({\mathbf{x}})]$$

17 $${{\varvec{\Psi}}} = \left[ {\begin{array}{*{20}c} {\sigma_{c}^{2} {{\varvec{\Psi}}}_{c} (X_{Bc} ,X_{Bc} )} & {\rho \,\sigma_{c}^{2} {{\varvec{\Psi}}}_{c} (X_{Bc} ,X_{Bf} )\,} \\ {\rho \,\sigma_{c}^{2} {{\varvec{\Psi}}}_{c} (X_{Bf} ,X_{Bc} )} & {\rho^{2} \sigma_{c}^{2} {{\varvec{\Psi}}}_{c} (X_{Bf} ,X_{Bf} ) + \sigma_{d}^{2} {{\varvec{\Psi}}}_{d} } \\ \end{array} } \right],$$

with ***M*** = [*ρ****M***_*c*_ ***M***_*d*_], moreover matrices (***F***_*c*_, *σ*_*c*_, ***Ψ***_*c*_, ***M***_*c*_) and (***F***_*d*_, *σ*_*d*_, ***Ψ***_*d*_, ***M***_*d*_) are derived, in turn, from ***s***_*KRc*_ and ***s***_*KRf*_^60^.

#### Design of experiments

**Figure 5:**
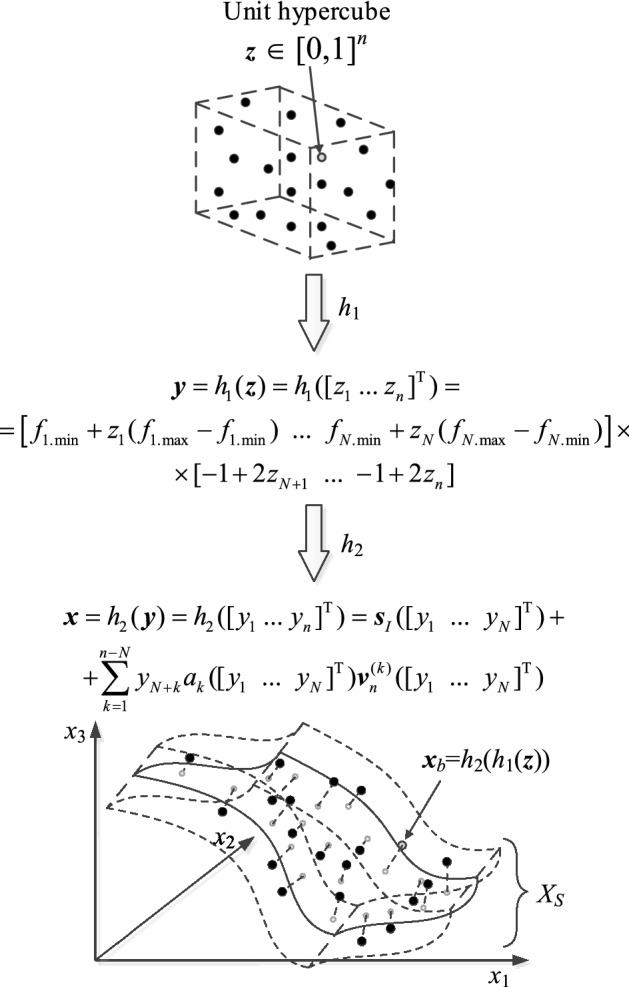
Conceptual illustration of the training data sampling process: (i) samples distributed according to Latin Hypercube Sampling (LHS)^81^ (top), (ii) mapping onto the Cartesian product of the performance figure space *F* and [−1,1]^*n*–*N*^ using function *h*_1_ (middle), (iii) black circles mark data samples projected onto the confined domain *X*_*S*_ using function *h*_2_ (bottom); gray circles indicate the samples distributed over the image of design space *F* prior to orthogonal extension.

A separate note should be made about the domain sampling procedure. Performing the design of experiments in a direct manner is inconvenient, as a geometry of the set *X*_*S*_ is relatively complex. Instead, we use the procedure explained in Fig. 5, which involves a surjective transformation between the unit cube [0, 1]^*n*^ and the constrained domain. According to this procedure, the samples ***x***_*B*_^(*k*)^ = *h*_2_(*h*_1_(***z***^(*k*)^)) ∈ *X*_*S*_ are uniformly distributed w.r.t. the objective space *F*, but not w.r.t. *X*_*S*_. The normalized samples {***z***^(*k*)^}, *k* = 1, …, *N*_*B*_, are distributed using Latin Hypercube Sampling, LHS^81^). Observe that the proposed modeling technique belong to a wider class of performance-driven (or constrained) modeling techniques, in which the surrogate is constructed within a region of the design space confined from the point of view of the design objectives. In contrast to the conventional modeling techniques, in performance-driven modeling, the surrogate domain constitutes a thin set within the classical deign space delimited by the lower and upper bounds on the design variables. As demonstrated in^82^, performing sequential sampling in a constrained domain does not bring any advantages over one-shot data sampling in terms of improving the model predictive power. This can be explained by a particular geometry of the constrained domain of the performance-driven surrogate, which encompasses nearly-optimum designs, the latter forming a manifold of a lower dimension than that of the original parameters space.

The function *h*_2_(*h*_1_()) also facilitates solving design tasks that involve the surrogate model, such as optimization. More specifically, the optimization process may be carried out over the unity cube [0, 1]^*n*^, where the design variables (i.e. geometry parameters of the antenna at hand) are transformed into the model domain for metamodel evaluation. Additionally, *s*_*r*_(***f***) can be used as an initial design of a high quality for any assumed performance figure vector ***f*** ∈ *F*.

It is expected that the incorporation of variable-fidelity models into the domain-confined surrogate, as described in this section, will lead to an additional computational savings over the method of^73^ with regard to the total costs of the model setup. This is because majority of the costs (related to the acquisition of both the trial and training points) will be reduced by the factor equal to the EM simulation time ratio between the models of low- and high-fidelity.

### Modeling procedure summary

**Figure 6:**
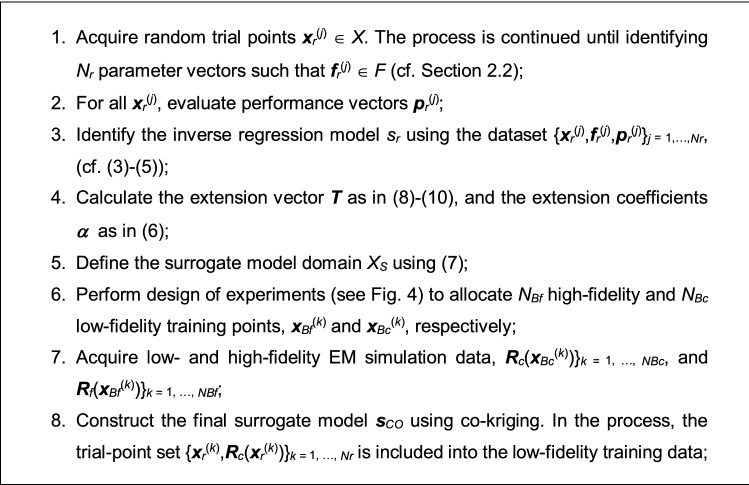
The workflow of the proposed antenna modeling procedure with variable-resolution EM simulations.

**Figure 7:**
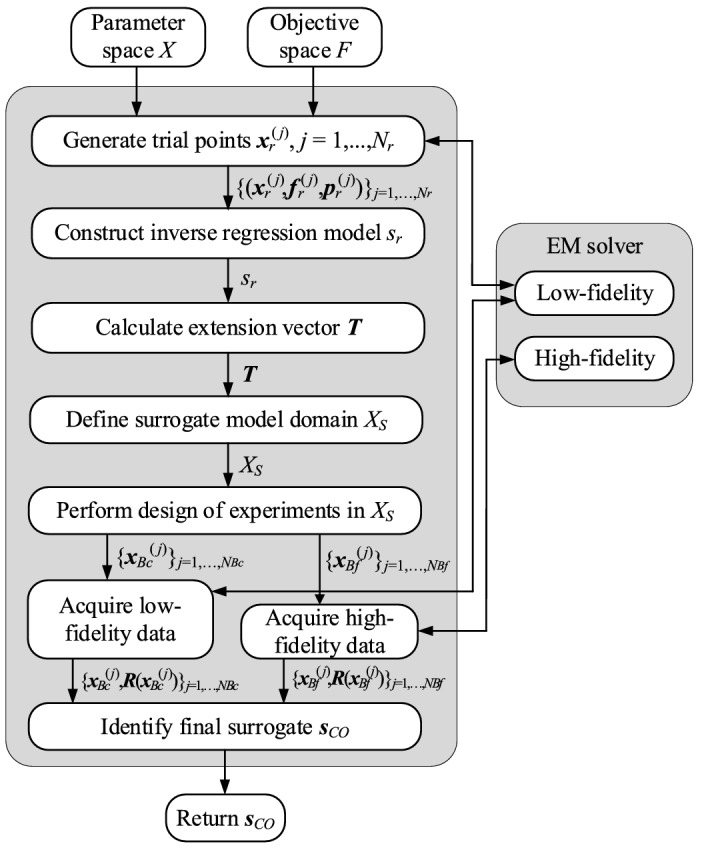
Operational flow of the presented two-layer modeling technique with domain confinement and variable-resolution EM simulations.

Here, we summarize the proposed modeling procedure, the components of which were described in sections performance-driven modeling basics through final surrogate. variable-resolution models and co-kriging. It should be noted that there is only one control parameter, the number of trial points *N*_*r*_, which is normally set to 50. The number of training data points (*N*_*Bf*_ of high-fidelity, and *N*_*Bc*_ of low-fidelity) depends on the required model accuracy. The modeling workflow has been provided in Fig. 6. Furthermore, Fig. 7 shows the flowchart of the process.

## Results

The modeling procedure introduced in section two-level variable-fidelity modeling within restricted domain has been validated in this section with the use of three exemplary microstrip antennas. The results obtained for various training data set sizes are compared to a number of benchmark methods including traditional (i.e. unconstrained) metamodels (kriging and RBF), along with the performance-driven frameworks (nested kriging^68^, and the reference-design-free approach^73^). The major figures of interest include the surrogates’ predictive power, its dependence on the cardinality of the training dataset, as well as the computational cost of the model setup.

### Verification antennas

**Figure 8:**
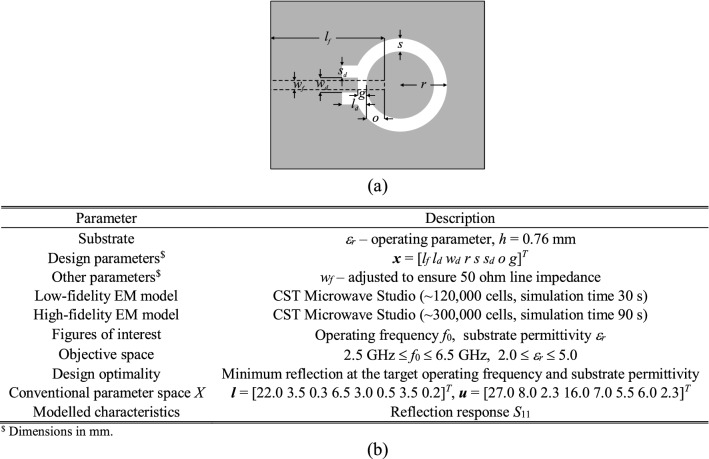
Antenna I: ring-slot microstrip antenna: (**a**) antenna geometry, (**b**) antenna parameters.

**Figure 9:**
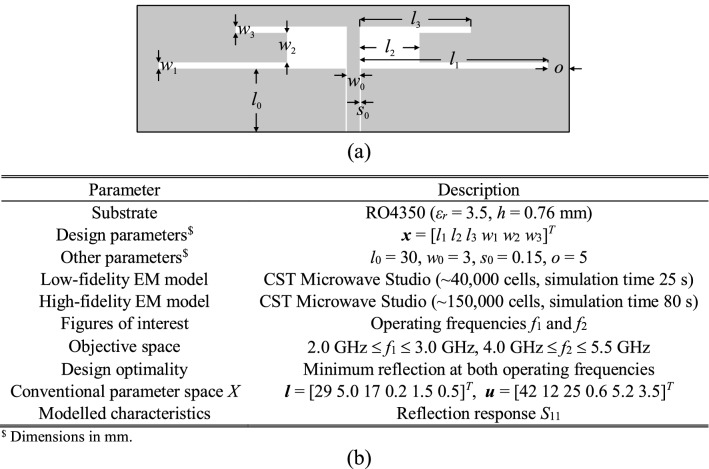
Antenna II: dual-band uniplanar dipole antenna: (**a**) antenna geometry, (**b**) antenna parameters.

**Figure 10:**
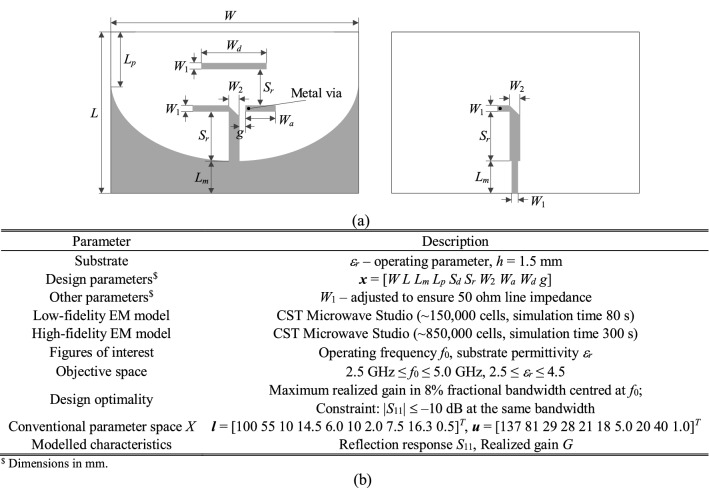
Antenna III: qasi-Yagi antenna with parabolic reflector: (**a**) antenna geometry, (**b**) antenna parameters.

**Figure 11:**
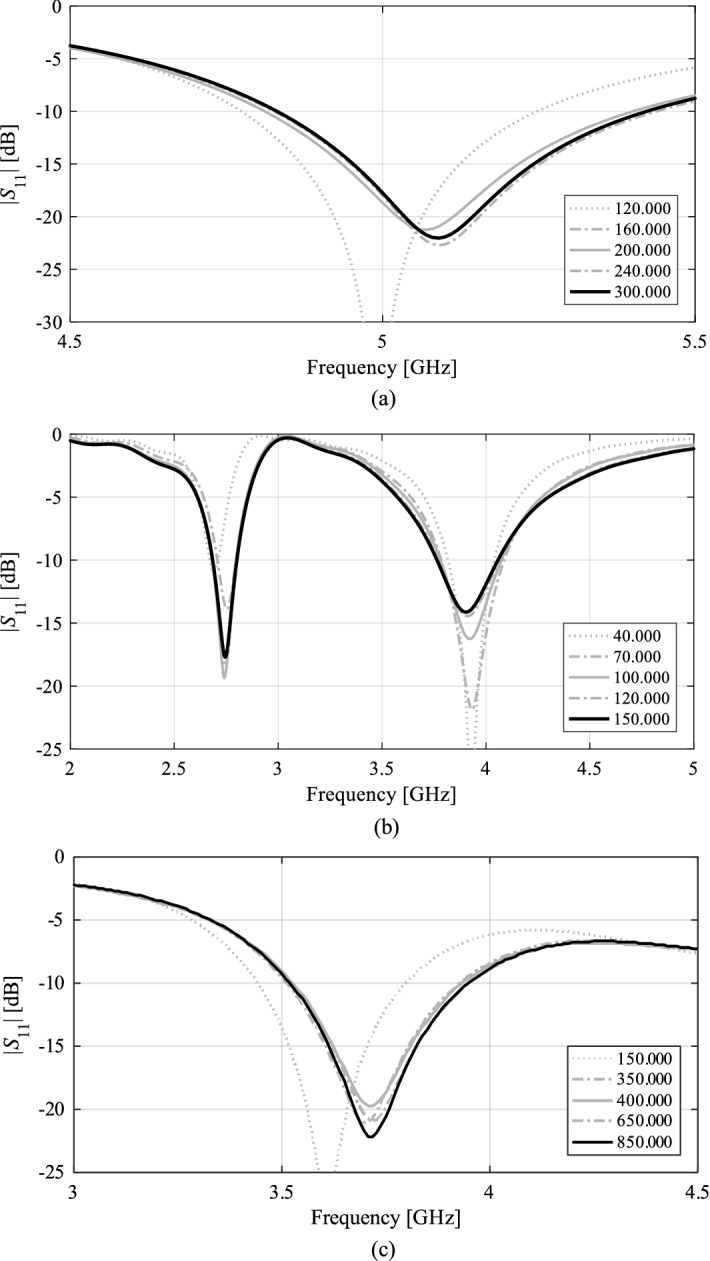
Grid convergence plots for: (**a**) Antenna I, (**b**) Antenna II, and (**c**) Antenna III for different numbers of mesh cells corresponding to various model fidelities from the lowest employed fidelity up to the highest one.

The antenna structures used for verification have been shown in Figs. 8, 9 and 10. These include a ring-slot antenna^83^, a dual-band dipole^84^, and a quasi-Yagi antenna with a parabolic reflector^85^, which henceforth are referred to as Antenna I, II and III, respectively. Figures 8, 9 and 10 also contain the relevant data, among others, designable parameters, operating parameters, and the details on the parameter and objective spaces. Furthermore, Fig. 11 shows the families of reflection responses corresponding to various model fidelities for all the benchmark antenna structures. Observe that all the benchmark antennas have been already validated, first, in their respective source papers^83–85^, and also in our previous work, e.g.^68,86^). Therefore, the experimental validation has not been provided, as being immaterial to the scope of the paper.

### Validation experiments setup

Antennas I, II and III have been modelled utilizing the technique proposed in this paper, as well as a number of benchmark methods, as listed in Table 2. The benchmark surrogates have been built using the training sets comprising 50, 100, 200, 400 and 800 data samples. The proposed models were obtained using several combinations of the coarse and fine datasets, in particular, *N*_*Bf*_ = 50 and *N*_*Bc*_ ∈ {50, 100, 200, 400, 800}, as well as *N*_*Bf*_ = 100 and *N*_*Bc*_ ∈ {50, 100, 200, 400, 800}. The cost of model construction is calculated in terms of the equivalent number of high-fidelity simulations, which considers the simulation time ratio of ***R***_*f*_ versus ***R***_*c*_. Also, the cost includes all additional expenses, e.g. those associated with the reference database designs acquisition for the nested kriging framework^68^, or generating the trial points for the method of^73^, and the introduced technique.

**Table 2:** Benchmark modeling methods.

Modeling approach	Domain	Setup
Kriging interpolation	Conventional (parameter space *X*)	Gaussian correlation function, second-order polynomial used as a trend function
Radial basis functions (RBF)	Conventional (parameter space *X*)	Gaussian correlation function, scaling coefficient determined through cross-validation
Nested kriging^81^	Confined domain *X*_*S*_	Thickness parameter *T* = 0.05 (Antennas I through III), 10 reference designs used in all cases, the numbers of EM simulations required for their identification: • Antenna I (acquisition cost 864 EM simulations) • Antenna II (acquisition cost 930 EM simulations) • Antenna III (acquisition cost 1899 EM simulations)
Reference-design-free modelling^65^	Confined domain *X*_*S*_	Thickness parameter *T* = 0.05 (Antennas I through III), the following numbers of EM simulation used to identify *N*_*r*_ = 50 accepted observables: • 106 (Antenna I) • 230 (Antenna II) • 192 (Antenna III)

A relative RMS error has been used as a measure of the model accuracy. It has been computed as ||***R***_*s*_(***x***)–***R***_*f*_(***x***)||/||***R***_*f*_(***x***)||, where ***R***_*s*_ and ***R***_*f*_ are the surrogate and EM-simulated antenna characteristics, respectively. The average errors are reported, which are obtained for one hundred independent testing points.

## Discussion

**Figure 12:**
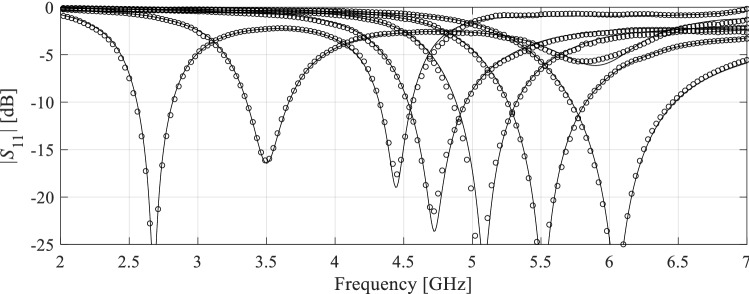
Antenna I: reflection responses at the representative test designs: full-wave model (—), and the presented two-level variable-fidelity surrogate (o) with *N*_*Bf*_ = 50 and *N*_*Bc*_ = 800.

**Figure 13:**
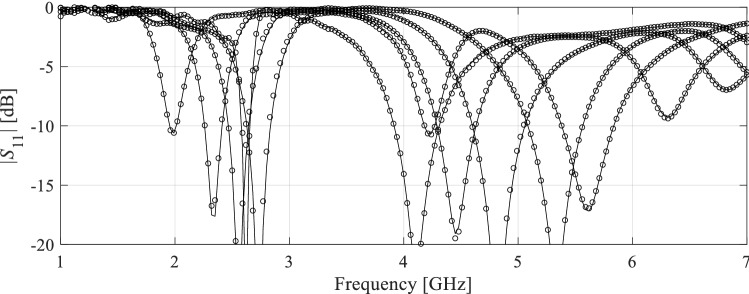
Antenna II: reflection responses at the representative test designs: full-wave model (—), and the presented two-level variable-fidelity surrogate (o) with *N*_*Bf*_ = 50 and *N*_*Bc*_ = 800.

**Figure 14:**
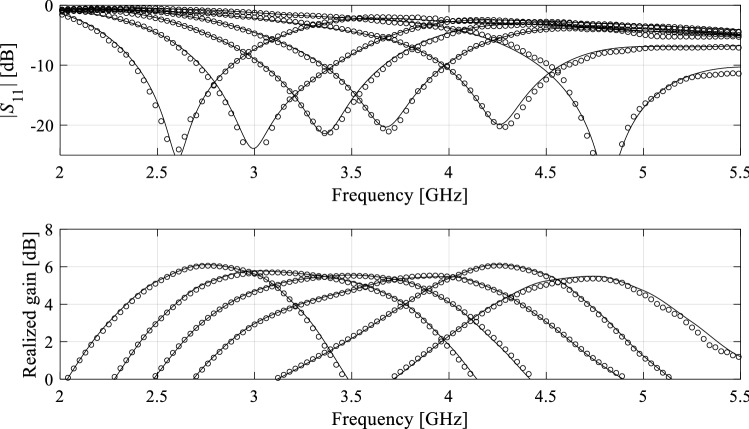
Antenna III: reflection responses at the representative test designs: full-wave model (—), and the presented two-level variable-fidelity surrogate (o) with *N*_*Bf*_ = 50 and *N*_*Bc*_ = 800.

The results can be found in Tables 3, 4 and 5, for Antenna I, II and III, respectively. Furthermore, Figs. 12, 13 and 14 illustrate the antenna responses at selected test locations, evaluated using the proposed model and EM simulation. The analysis of the data in the tables leads to the following observations:

- The accuracy of the presented model outperforms that of the conventional (i.e. unconstrained) models. In particular, for Antennas I and III, the unconstrained surrogates are unable to yield the surrogates of usable accuracy: the modeling error is still beyond 25% even for the training sets of the largest sizes.
- The predictive power of the introduced surrogate is also better than for the nested kriging metamodel^68^ set up using smaller training data sets, which is due to the inclusion of the random trial points into the training dataset. For larger values of *N*_*B*_, the models exhibit similar accuracy. This is also the case for the reference-design-free method, the accuracy of which is comparable to the proposed model due to sharing the rules for model domain definition. Yet, for the smaller training sets, the proposed model is more accurate, which is because the overall number of samples (low- and high-fidelity together) is larger than for the method of^73^. Perhaps the most important advantage of the proposed methodology is an excellent computational efficiency. Executing majority of the operations with the use of low-fidelity model leads to considerable savings, as reported in Tables 3, 4 and 5. For example, the cost reduction over conventional surrogates is as high as 55, 54 and 60 percent for Antennas I, II and III, respectively, assuming *N*_*B*_ = 800 and *N*_*Bf*_ = 50.
- The savings over the nested kriging model are even higher due to the extra expenses entailed by the reference design acquisition in^68^. We have 78, 79 and 88 percent savings for Antennas I through III, respectively, also for *N*_*B*_ = 800 and *N*_*Bf*_ = 50. The savings over the reference-design-free methods^73^ are 61, 64 and 68 percent, for Antenna I, II and III, respectively, again assuming *N*_*B*_ = 800 and *N*_*Bf*_ = 50. It should be reiterated that the cost reduction with respect to the benchmark performance-driven methods does not deteriorate the modeling accuracy.

**Table 3:** Antenna I: modeling results and benchmarking.

Modeling method	Number of training samples^e^				
	50	100	200	400	800
**Kriging**					
Modeling error	56.9%	50.8%	35.8%	31.5%	25.6%
Model setup cost^b^	50	100	200	400	800
Modeling error	61.0%	53.2%	37.9%	34.1%	27.2%
**RBF**					
Model setup cost^b^	50	100	200	400	800
Modeling error	19.4%	12.9%	7.7%	5.1%	3.7%
**Nested kriging**^68^					
Model setup cost^a^	914	964	1064	1264	1664
Modeling error	13.4%	9.9%	6.9%	5.4%	4.4%
**No-reference-design modeling**^73^					
Model setup cost^c^	156	206	306	506	906
Modeling error	13.1%	8.5%	6.7%	5.1%	4.3%
**Two-stage variable-resolution surrogate (this work)**					
***N***_***Bf***_** = 50**					
Model setup cost^d^	106.7	123.3	156.7	223.3	356.7
Modeling error	8.2%	7.7%	5.3%	4.2%	3.5%
***N***_***Bf***_** = 100**					
Model setup cost^d^	156.7	173.3	206.7	273.3	406.7

^a^The cost includes acquisition of the reference designs, which is 864 EM high-fidelity simulations of the antenna when using feature-based optimization^67^ as listed in the table. Conventional (minimax) optimization required high-fidelity 1012 simulations.

^b^The cost refers to high-fidelity simulations.

^c^The cost includes generation of random trial points, here, 106 high-fidelity simulations in total to yield *N*_*r*_ = 50 accepted samples.

^d^The cost includes generation of random trial points at low-fidelity level, here, 120 simulations in total to yield *N*_*r*_ = 50 accepted samples.

^e^In the case of the proposed model, the numbers 50 through 800 refer to low-fidelity EM simulations.

**Table 4:** Antenna II: modeling results and benchmarking.

Modeling method	Number of training samples^e^				
	50	100	200	400	800
**Kriging**					
Modeling error	21.7%	17.3%	12.6%	9.3%	7.2%
Model setup cost^b^	50	100	200	400	800
Modeling error	24.9%	19.8%	14.3%	10.5%	8.7%
**RBF**					
Model setup cost^b^	50	100	200	400	800
Modeling error	9.9%	6.4%	4.4%	3.8%	3.4%
**Nested kriging**^68^					
Model setup cost^a^	980	1030	1130	1330	1730
Modeling error	7.3%	5.1%	3.8%	3.1%	2.5%
**No-reference-design modeling**^73^					
Model setup cost^c^	280	330	430	630	1,030
Modeling error	5.4%	3.1%	2.5%	2.1%	1.7%
**Two-stage variable-resolution surrogate (this work)**					
***N***_***Bf***_** = 50**					
Model setup cost^d^	132.8	148.4	179.7	242.2	367.2
Modeling error	3.5%	3.0%	2.3%	1.8%	1.6%
***N***_***Bf***_** = 100**					
Model setup cost^d^	182.8	198.4	229.7	292.2	417.2

^a^The cost includes acquisition of the reference designs, which is 930 EM high-fidelity simulations of the antenna when using feature-based optimization^67^ as listed in the table. Conventional (minimax) optimization required 1201 high-fidelity simulations.

^b^The cost refers to high-fidelity simulations.

^c^The cost includes generation of random observables, here, 230 high-fidelity simulations in total to yield *N*_*r*_ = 50 accepted samples.

^d^The cost includes generation of random trial points at low-fidelity level, here, 215 simulations in total to yield *N*_*r*_ = 50 accepted samples.

^e^In the case of the proposed model, the numbers 50 through 800 refer to low-fidelity EM simulations.

**Table 5:** Antenna III: modeling results and benchmarking.

Modeling method	Number of training samples^e^				
	50	100	200	400	800
**Kriging**					
Modeling error	61.4%	50.7%	39.8%	32.8%	31.8%
Model setup cost^b^	50	100	200	400	800
**RBF**					
Modeling error	65.3%	51.8%	43.2%	37.1%	33.6%
Model setup cost^b^	50	100	200	400	800
**Nested kriging**^68^					
Modeling error	17.9%	13.3%	7.5%	5.4%	4.5%
Model setup cost^a^	1949	1999	2099	2299	2699
**No-reference-design modeling**^73^					
Modeling error	10.8%	8.4%	7.1%	5.9%	5.0%
Model setup cost^c^	242	292	392	592	992
**Two-stage variable-resolution surrogate (this work)**					
***N***_***Bf***_** = 50**					
Modeling error	8.7%	7.2%	6.7%	6.5%	6.0%
Model setup cost^d^	118.5	131.9	158.5	211.9	318.5
***N***_***Bf***_** = 100**					
Modeling error	6.8%	6.1%	5.4%	5.3%	5.2%
Model setup cost^d^	168.5	181.9	208.5	261.9	368.5

^a^The cost includes acquisition of the reference designs, which is 1899 EM high-fidelity simulations of the antenna.

^b^The cost refers to high-fidelity simulations.

^c^The cost includes generation of random trial points, here, 192 high-fidelity simulations in total to yield *N*_*r*_ = 50 accepted samples.

^d^The cost includes generation of random trial points at low-fidelity level, here, 207 simulations in total to yield *N*_*r*_ = 50 accepted samples.

^e^In the case of the proposed model, the numbers 50 through 800 refer to low-fidelity EM simulations.

## Conclusion

This work presented a novel framework for surrogate modeling of antenna structures. Our approach exploits a constrained modeling paradigm with the surrogate model domain established using random trial points and the operating parameter data extracted therefrom. Furthermore, variable-resolution EM models are used at the domain definition stage and the surrogate construction phase. The latter combines sparsely acquired data samples of high-fidelity with densely collected low-fidelity training points. The final surrogate is rendered using co-kriging. Extensive numerical experiments confirm that the proposed method outperforms conventional methods. Furthermore, the predictive power of the presented surrogate is comparable or slightly better than for the benchmark performance-driven models. In terms of the computational efficiency, our modeling framework by far outperforms all the techniques employed in the numerical studies, both the conventional and the performance-driven ones. The computational savings over the traditional models are as high as 56 percent on the average, whereas they are 82 and 64 percent over the nested kriging modeling technique and the database-design-free method, respectively. The procedure presented in this work can be considered a viable alternative to state-of-the-art modeling routines, especially for more demanding conditions that include multi-dimensional spaces with broad geometry and material parameters’ ranges, the same pertains to the operating conditions.

## Data Availability

The datasets generated during and/or analysed during the current study are available from the corresponding author on reasonable request.
